# Real-Life Multicenter Experience of Venetoclax in Combination with Hypomethylating Agents in Previously Untreated Adult Patients with Acute Myeloid Leukemia in Greece

**DOI:** 10.3390/jcm13020584

**Published:** 2024-01-19

**Authors:** Theodora Chatzilygeroudi, Ismini Darmani, Natali El Gkotmi, Pinelopi Vryttia, Stavroula Douna, Anthi Bouchla, Vasiliki Labropoulou, Maria Kotsopoulou, Argiris Symeonidis, Maria Pagoni, Vasiliki Pappa, Sotirios G. Papageorgiou

**Affiliations:** 1 Hematology Division, Department of Internal Medicine, School of Health Sciences, Faculty of Medicine, University of Patras, 30100 Patras, Greece; thchatzilygeroudi@gmail.com (T.C.); vas.labropoulou@gmail.com (V.L.); argiris.symeonidis@yahoo.gr (A.S.); 2 Hematology Department, Evaggelismos General Hospital, 10676 Athens, Greece; isminidarmani@gmail.com (I.D.); natelgkotmi@gmail.com (N.E.G.); marianpagoni@yahoo.com (M.P.); 3 Hematology Unit, Second Department of Internal Medicine, and Research Institute, Medical School, University General Hospital “Attikon”, National and Kapodistrian University of Athens, 12462 Athens, Greece; pinelopivrt@gmail.com (P.V.); anthibouhla@hotmail.com (A.B.); vas_pappa@yahoo.com (V.P.); 4 Hematology Department, Metaxa General Hospital, 18537 Peiraeus, Greece; stavria.douna@gmail.com (S.D.); mariakotsopoulou@gmail.com (M.K.)

**Keywords:** AML, frontline treatment, hypomethylating agents, HMAs, venetoclax, real-world data

## Abstract

Background: The landscape of first-line treatment for acute myeloid leukemia (AML) patients ineligible for intensive chemotherapy has changed remarkably after venetoclax approval. Accumulating real-world data further apprises us with more knowledgeable use. To assess the efficacy and safety challenges in the real-life setting of the combination of hypomethylated agent (HMA) and venetoclax, we conducted a multi-center retrospective study. Methods: Forty adult AML patients treated with the combination of HMA and venetoclax as a first-line treatment after full approval (2020) were included. To confirm VIALE-A results, this group was compared to a historical cohort of 17 chemotherapy-ineligible AML patients treated with HMA monotherapy before 2020. Results: The combination of HMA-venetoclax achieved a composite complete response rate of 86.8% (*p* < 0.001), median overall survival, and event-free survival of 33.8 and 19.7 months, respectively, in a median follow-up of 17.8 months (p_os_ < 0.001, HR = 0.276, CI: 0.132–0.575, p_EFS_ = 0.004, HR = 0.367, CI: 0.174–0.773). High rates of neutropenia (90%) and consequent infection rates (57.5%) were noted. Only 55% of our patients received antifungal prophylaxis, as its use remains controversial, and invasive fungal infections were presented in 7.5%. Conclusions: Evidently, venetoclax-HMA yields high response rates and profound survival benefits in real life and has changed our approach to alternative chemotherapy options.

## 1. Introduction

Treatment of elderly or unfit patients with acute myeloid leukemia (AML) has changed since the FDA’s (Food and Drug Administration) approval of venetoclax-based regiments in 2018 [1,2]. Before full approval of venetoclax combinations in 2020, hypomethylating agent (HMA) monotherapy was the standard of care for non-eligible intensive chemotherapy AML patients, resulting in a complete response (CR) and CR with an incomplete count recovery (CRi) rate of only 15–30% and a median overall survival (OS) of less than a year [3,4]. 

Venetoclax is an inhibitor of the B-cell leukemia/lymphoma-2 (BCL-2) anti-apoptotic protein, which is overexpressed in leukemia stem cells (LSCs) [5], and when administered with HMAs, disrupts their metabolic machinery, driving energy metabolism and eradicating LSCs [6]. An open-label, phase 1b study of the HMA-venetoclax combination demonstrated CR + CRi > 70% and median OS of 16.9 and 16.2 months for azacytidine and decitabine, respectively [2]. These results were confirmed with a CR + CRi rate of 66.4% in VIALE-A, and full approval was obtained [7]. Following this, the combination of HMAs with venetoclax has been increasingly used over the last 3 years as a first-line treatment for patients non-eligible for intensive chemotherapy and allogeneic stem-cell transplantation (all-SCT) [8], translating into accumulating real-world data [9,10,11,12,13,14].

In these first years of venetoclax use, many challenges about its use in everyday practice have arisen [15,16]. Venetoclax-induced myelosuppression often requires dose modifications and the use of granulocyte-colony stimulating factor (GCSF) [15,17]. Even though prolonged neutropenia would justify the need for antifungal prophylaxis, drug interactions with CYP3A4 inhibitors and the consequent necessity for dose reduction of venetoclax raise concerns about drug underexposure [18].

Conducting a retrospective analysis in 4 different centers in Greece, we aimed to determine efficacy, toxicity, and survival outcomes for the HMA-venetoclax combination as a first-line treatment for AML in an everyday clinical setting. Moreover, we assessed CYP3A4 inhibitors’ and particularly antifungal use in our centers, as well as dose modifications of venetoclax during treatment. Lastly, we compared results to HMA monotherapy-treated AML patients in recent years before venetoclax approval in our country and assessed differences in patient populations selected for each treatment approach.

## 2. Materials and Methods

A total of fifty-seven adult patients with de novo AML non-eligible for chemotherapy from 4 centers were included in our study, diagnosed between July 2018 and August 2023. Seventeen of them received HMA monotherapy as first-line treatment before the 2020 full approval of venetoclax, and 40 were treated with the HMA-venetoclax combination. Azacitidine 75 mg/m^2^ on days 1–7 or decitabine 20 mg/m^2^ on days 1–5 were administered in all centers. This study was conducted in accordance with the Declaration of Helsinki.

Bone marrow and peripheral blood baseline blast count, basic cytogenetic data, and molecular data, such as G-banding karyotype, polymerase chain reaction (PCR) testing for NPM1 and FLT3 status, and next-generation sequencing data (NGS) for gene mutational status were collected. Moreover, comorbidity status at diagnosis, concomitant medications, demographics, and performance status data were reviewed retrospectively. The Karnofsky scale score was documented to assess performance status, and the Cumulative Illness Rating Scale-Geriatric (CIRS-G) score was evaluated from comorbidity documentation. 

Response and risk stratification were assessed according to European Leukemia Net (ELN) 2017 criteria [19], and the time of the first evaluation was recorded. Composite CR (CCR) included CR_mrd−_, CR, CR_i,_ and morphological leukemia-free state (MLFS). Treatment delay or dose reduction was assessed for both HMAs and venetoclax. Ramp-up scheduling and the final dose of venetoclax were recorded. Tumor lysis syndrome (TLS) risk was defined as low for patients with white blood cell count (WBC) < 25 × 10^9^/L and LDH < 2× upper limits of normal (ULN) value, intermediate for patients with WBC < 25 × 109/L and LDH ≥ 2 × ULN or 25 × 10^9^/L ≤ WBC < 100 × 10^9^/L, and high for patients with WBC ≥ 100 × 10^9^/L at diagnosis [20].

Pneumocystis jirovecii (PCP), antiviral, and antifungal prophylaxis were reviewed. The use of posaconazole and other strong or moderate CYP3A4 inhibitors was assessed. Hematological toxicity and GCSF use, as well as the incidence of TLS, febrile neutropenia, and infection, were reviewed. 

OS was evaluated from the date of diagnosis. Event-free survival (EFS) was estimated from the date of treatment initiation to the date of primary refractory disease, relapse, or death of any cause. Relapse-free survival (RFS) was estimated from the date of the first response to the date of relapse or death of any cause for patients with CCR. The OS, EFS, and RFS were evaluated by the Kaplan–Meier method, and differences between the two groups were assessed with the log-rank test. To determine differences between groups, the chi-square test or Fisher’s exact test was used for nominal data, and the Student’s *t*-test or Mann–Whitney U test was used for continuous variables, depending on the Kolmogorov–Smirnov test for normality of the data. A Cox proportional hazard model analysis was performed to identify predictors of shortened OS and RFS. The *p* values < 0.05 were considered statistically significant. Analyses were conducted with SPSS v28.0.

## 3. Results

### 3.1. Patient Characteristics

Forty de novo AML patients with a median age of 73 years (range: 52–87) received a combination of HMA and venetoclax as a first-line treatment. Of note, more than half (23/40) of the patients in this group were younger than 75 years old. The corresponding median age for the HMA monotherapy group was significantly older: 76 years (range: 64–86), (*p* = 0.004). As far as comorbidities are concerned, 55% of the patients in the combination group had cardiovascular disease at diagnosis, 42.5% had dyslipidemia, 25.6% had thyroid disease, and almost 20% had arrythmia. Patients had a median CIRS-G score of 8 (range: 4–12) and a Karnofsky Performance Scale score median value of 90 (range: 60–100). Even though there was no difference between the two groups concerning CIRS-G score (*p* = 0.249), there was a trend for higher rates of cardiovascular disease (*p* = 0.125), congestive heart failure (*p* = 0.085), diabetes (*p* = 0.093), and hyperlipidemia (*p* = 0.145) in the HMA monotherapy group. Karnofsky Performance Scale score evaluation revealed a significant difference (*p* < 0.001) in performance status between the two groups, with a median value of 50 (range: 20–100) in the HMA monotherapy group (Table 1). 

A percentage of 22.5% of patients presented with leukocytosis ≥ 25 × 10^9^/L in the combination group, whereas moderate or severe anemia (Hb < 10 g/dL) and thrombocytopenia (<100 × 10^9^/L) were present in 87.5% and 62.5% of patients at diagnosis, respectively. Presentation with leukocytosis ≥ 25 × 10^9^/L was equivalent in the two groups (*p* = 0.176), and cytoreduction was used in 17.6% of the patients in the monotherapy arm and in 20% of patients in the combination arm before initiating treatment (*p* = 0.576). Anemia and thrombocytopenia at diagnosis did not differ between the two groups. In the combination group, the median bone marrow blast count at diagnosis was 40%. The peripheral blood blast count was only recorded in 23/40 patients, and the median value was 17%. Only one patient with extramedullary disease was included in this group. The TLS risk score was low in 62.5%, intermediate in 20%, and high in the remaining 5% of the combination treatment patients. 

As for HMA treatment, azacitidine was mostly selected between the two HMAs for monotherapy treatment (64.7%), and the vast majority of our venetoclax combination-treated cohort (82.5%) received azacytidine as well. Venetoclax was administered with the known schedule of ramp-up initial dosing in 87.5% of the cases, and the final dose was 400 mg in 75% of the cases and 12.5% each of 200 mg or 100 mg. 

ELN 2017 risk stratification, cytogenetic risk, NPM1 and FLT3 status, and NGS data for both treatment groups are presented in Table 1, along with the rest of the baseline patient and disease characteristics. 

### 3.2. Prophylaxis and Toxicity

Hematologic toxicity in our cohort of HMA-venetoclax-treated patients was very common (Table 2). Neutropenia was the most common side effect (90%) and was in most cases grade 3 and 4, followed by anemia (Hb < 11 g/dL) and thrombocytopenia (<100 × 10^9^/L) that were observed in 85% and 67.5%, respectively. Compared with the combination group, a lower frequency of neutropenia (64.7%; *p* = 0.020) was documented in the HMA monotherapy group. Anemia and thrombocytopenia were noted in this group at a rate of 66.7% and 52.9%, respectively (Table 2).

In our cohort, GCSF was used in 80% (32/40 patients) of the cases during HMA-venetoclax treatment, even in three patients that did not achieve CR. This rate was not different from that observed in the monotherapy group at 60% (10/17 patients) (*p* = 0.113). Delay or reduction in administration of venetoclax was observed in the vast majority (87.5%) and definite discontinuation in 20% of combination-treated patients. Correspondingly, for concomitant HMA treatment, delay was observed in 60% of patients and discontinuation in 12.5% of patients. Delay or reduction of HMA in the HMA monotherapy treatment setting occurred in 41.2%, similarly to the combination-treated patients (*p* = 0.366).

Well-documented infections and febrile neutropenia were noted in 23 (57.5%) and 27 patients (67.5%) in the combination group, respectively. Infections were mostly lower respiratory tract infections (11/23, 47.8%), followed by urinary tract infections (5/23, 21.7%), bacteriemia (5/23, 21.7%), COVID-19 (4/27, 14.8%), fungal infection (3/23, 13%), clostridium difficile infection (3/23, 13%), and tuberculosis (1/23, 7.6%).

Documented infections were noted at equivalent rates (52.9% for the HMA monotherapy group, *p* = 0.501), and febrile neutropenia was also present in half of the monotherapy-treated patients (*p* = 0.322). Infections were mostly lower respiratory tract infections as in the combination group, whereas fungal infections were not documented and bacteriemia presented in only one patient in the HMA monotherapy group. It is of interest that antifungal prophylaxis was used for more than half (58.8%) of the HMA monotherapy-treated patients, and the most common choices of antifungal were posaconazole (5/10) and fluconazole (5/10).

In the venetoclax combination arm, antiviral and PCP prophylaxis were given at 32.5% and 12.5%, respectively. Moreover, strong and moderate inhibitors of CYP3A4 were administered to 22.5% of the patients, and p-glycoprotein inhibitors to 7.5%. Antifungal prophylaxis was used in more than half of our patients (55%), but posaconazole was only used in 18% of the cases that received prophylaxis and 10% of the full cohort of patients receiving HMA-venetoclax. Other antifungals used as prophylaxis were micafungin (27.2%), amphotericin B (13.6%), anidulafungin (9%) in hospitalized patients, and fluconazole (45.5%) in outpatients. For one patient receiving posaconazole, severe hepatotoxicity occurred. Other rare adverse events observed in only one patient were Sweet syndrome, arthralgia, dyspnea, arrythmia, and headache. 

Moreover, TLS occurred in 4/40 patients (10%), even though cytoreduction (2/4 patients) and ramp-up of venetoclax dosing (4/4 patients) were used to avoid it. Of note, only one of these patients had a high TLS score. 

### 3.3. Response Evaluation and Outcome

In the combination arm, patients received a median of 6 cycles (range: 1–35), while the median time to first assessment was 1.08 months, significantly shorter than the HMA monotherapy-treated patients (4.82 months; *p* < 0.001). 

The vast majority of the patients in the HMA-venetoclax group (38/40) had a response evaluation available. One patient was not yet evaluated after 4 months of treatment. CCR was achieved in 33 (86.8%) patients, CR and CRi in 10 (26.3%) and 20 (52.6%) patients, respectively, and MLFS in 1 patient. CR_mrd−_ was documented only in two patients (5.2%). Five of our patients (13.1%) did not respond to treatment (NR). Of the 32 patients (84.2%) with CR_mrd−_/CR/CRi, 10 presented with an abnormal karyotype at diagnosis, 9 had NPM1, and 4 FTL3 mutated AML. One-third of patients achieving CR_mrd−_/CR/CRi were tested with NGS at diagnosis, and all harbored one or more mutations.

In the HMA monotherapy group, four out of seventeen (23.5%) patients were not evaluated for response due to having an OS of less than six months. CCR, mostly evaluated by MLFS, was achieved in 4 out of 13 evaluated patients (30.8%), significantly less than in HMA-venetoclax-treated patients (*p* < 0.001). Eight of the HMA-monotherapy-treated patients (61.5%) did not respond to treatment (NR), and 1/13 achieved a partial response (PR), as shown in Table 3. 

**Figure 1:**
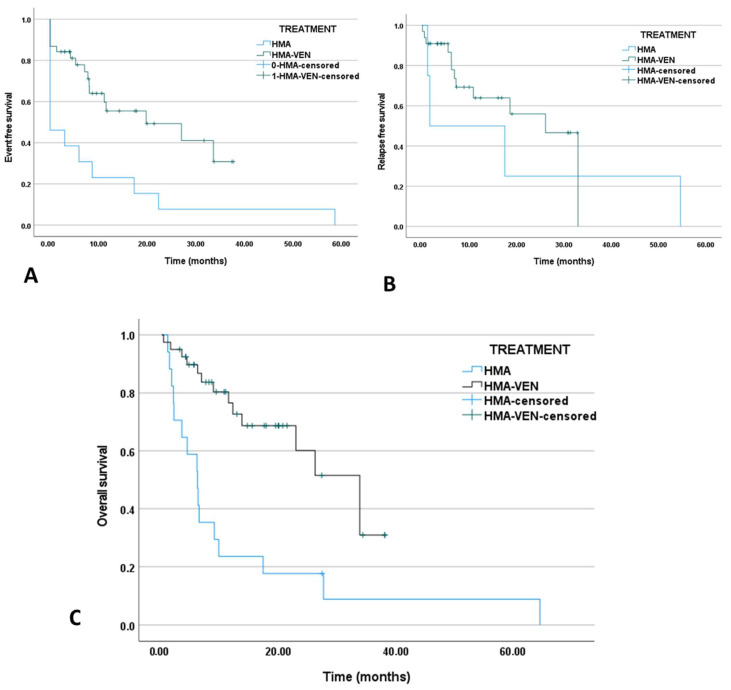
(**A**) EFS of HMA-monotherapy-treated versus HMA-venetoclax combination-treated AML patients (p_EFS_ = 0.004, HR = 0.367, CI: 0.174–0.773). (**B**) RFS of HMA monotherapy versus HMA-venetoclax-treated AML patients (p_RFS_ = 0.492, HR = 0.631, CI: 0.168–2.3). (**C**) OS of HMA monotherapy versus HMA-venetoclax-treated AML patients (p_OS_ < 0.001, HR = 0.276, CI: 0.132–0.575).

After a median follow-up time of 17.8 months, 14 (35%) patients had died, with causes of death being progressive disease (*n* = 7), infection (*n* = 6), hemorrhagic complications (*n* = 2), and TLS (*n* = 1). Early mortality of less than 30 days was presented in one patient in our cohort who died from a hemorrhagic event. The median EFS was estimated at 19.7 months, and the median RFS for the combination group was 26.1 months (range: 0.1–33 months) (Figure 1A,B). Ten of the thirty-three (30.3%) responding patients experienced a relapse. Four out of ten relapsed patients received intensive chemotherapy (40%) as a salvage treatment, and other second-line treatment options involved swapping azacitidine with decitabine or replacing the HMA with the IDH1 inhibitor ivosidenib. The median OS for the complete cohort of HMA-venetoclax-treated patients was 33.8 months (Figure 1C), and separate analyses showed 34.1 months and 6.7 months for responders and non-responders, respectively. 

For our cohort of HMA-monotherapy-treated patients, after a median follow-up time of 6.1 months, 16/17 patients died, and causes of death included progressive disease (*n* = 5) and infection (*n* = 10). Of note, two toxic HMA-related deaths were observed. Median EFS was estimated to be 0 months, reflecting poor response documented outcomes, evidently lower than the HMA-venetoclax combination treated patients (p_EFS_ = 0.004, HR = 0.367, CI: 0.174–0.773). Moreover, for the few responding patients, median RFS was evaluated at only 1.64 months (p_RFS_ = 0.492, HR: 0.631, CI: 0.168–2.36). Second-line treatments included intensive chemotherapy (33.3%), venetoclax-based treatment (22.2%), and ivosidenib (22.2%). The median OS was 6.1 months, significantly lower than the HMA-venetoclax-treated group (p_OS_ < 0.001, HR = 0.276, CI: 0.132–0.575). Kaplan–Meier curves with prominent benefits for EFS and OS for the HMA-venetoclax combination regiment are exhibited in Figure 1. 

To identify prognostic factors for OS and RFS, Cox regression analysis was conducted, and the variables assessed are shown in Table 4. Receiving less than the standard dose (400 mg) of venetoclax did not have a significant prognostic impact on either RFS or OS. Moreover, even though delay or reduction of venetoclax, as well as the use of CYP3A4/P-gp inhibitors, did not have any prognostic value, definite discontinuation of venetoclax was demonstrated to be predictive of shorter RFS and OS (p_RFS_ = 0.01, HR: 7.23, CI: 1.6–32.65, and p_OS_ = 0.006, HR: 5.38, CI: 1.64–20.72). Lastly, even though a high ELN risk score had no prognostic value for OS, it was shown to predict shorter RFS (*p* = 0.021, HR: 4.98, CI: 1.27–19.47). Upon multivariate analysis, both adverse ELN risk and discontinuation of venetoclax preserved predictive value for shorter RFS (p_ELN_ = 0.038, HR: 4.33, CI: 1.085–17.27, p_stopVEN_ = 0.045, HR: 4.87, CI: 1.036–22.92). 

**Figure 2:**
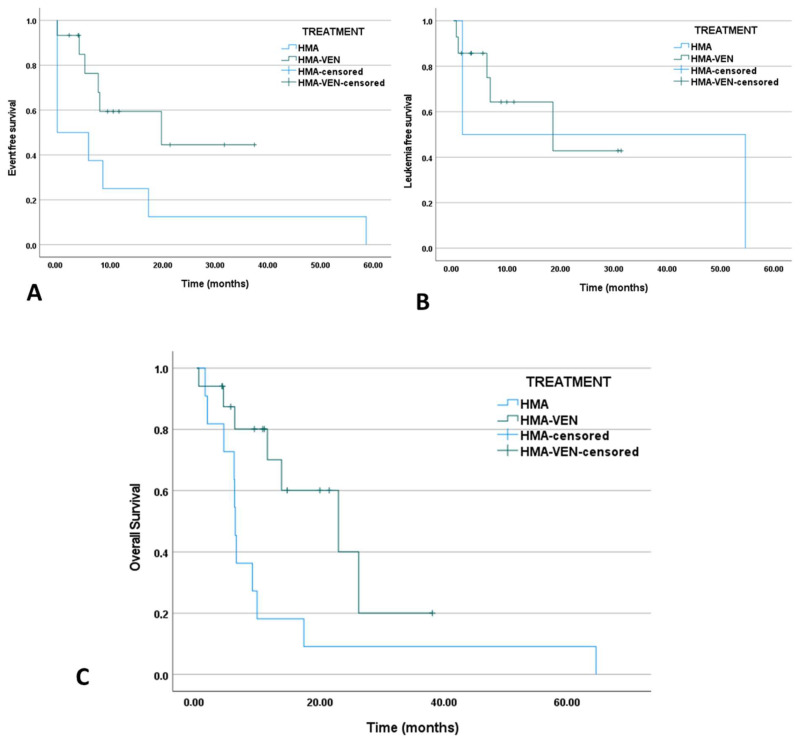
(**A**) Event-free survival of HMA-monotherapy-treated versus HMA-venetoclax combination-treated AML patients older than 75 years old (elderly) (p_EFS_ = 0.055, HR = 0.371, CI: 0.124–1.11). (**B**) RFS of HMA monotherapy versus HMA-venetoclax-treated elderly AML patients (p_RFS_ = 0.907, HR = 0.878, CI: 0.101–0.767). (**C**) OS of HMA monotherapy versus HMA-venetoclax-treated elderly AML patients (p_OS_ = 0.02, HR = 0.329, CI: 0.124–0.876).

After eliminating patients younger than 75 years old from both treatment groups, the CCR rate remained significantly higher (*p* < 0.001). Median EFS for HMA-venetoclax-treated patients over 75 years of age was 19.7 months versus 0 months for the HMA monotherapy-treated (*p* = 0.055, HR = 0.371, CI: 0.124–1.11). Moreover, the median RFS of responders was evaluated at 1.64 months for HMA-monotherapy-treated patients versus 18.59 months for HMA-venetoclax-treated patients (p_RFS_ = 0.908, HR: 0.88, CI: 0.101–7.67). Additionally, a significant OS benefit for HMA-venetoclax-treated patients in this age group was demonstrated in accordance with VIALE-A, with a median OS of 22.9 months versus 6.2 months for the HMA monotherapy-treated cohort (*p* = 0.02, HR = 0.329, CI: 0.124–0.876). Kaplan–Meier curves for this age group are exhibited in Figure 2A–C.

## 4. Discussion

The evolution of the treatment strategy for elderly or unfit patients with AML after venetoclax approval is unquestionable. From our analysis, as well as many other real-life data, it is evident that the high CR rates of the HMA-venetoclax regiment may justify its use in younger populations as well [12,14,21,22]. The difference in Karnofsky scores between the two compared groups implies that there was a specific therapeutic gap filled with venetoclax approval for patients not fit enough at the time of diagnosis for chemotherapy, but not old enough to be given only a 15–30% chance of response with HMA alone treatment [3,4]. This is also underlined by the fact that 30% of non-responding patients treated with HMA alone before venetoclax received full approval were offered intensive chemotherapy due to a lack of alternative options. Interestingly, in our combination cohort, 4 out of 10 patients who relapsed were able to receive salvage chemotherapy, indicating that their performance status may have improved with combination treatment.

The marginal survival benefit of less than 8 months [23] and low CR rates of HMAs in elderly or unfit AML patients [3,23] were confirmed by our study, and a dramatic amelioration in response rates was observed in patients receiving HMA combined with venetoclax after its approval in 2020 (*p* < 0.001). Even after eliminating younger than 75-year-old patients, the difference in response rates remained prominent (*p* < 0.001). The CCR rate of HMA-venetoclax in our study was 86.8%, confirming the previously reported good outcomes [7]. Even though recent meta-analyses for HMA-venetoclax in newly diagnosed AML in a real-life setting indicate lower OS [10], in our cohort, the median OS of patients older than 75 years was 22.9 months, even higher than VIALE-A survival of 17.5 months [7]. When only younger patients were included, OS exceeded 2 years (33.8 months). EFS for the full cohort of HMA-venetoclax-treated patients, as well as the elderly group, was estimated at 19.7 months, more than the 16.3 months documented in VIALE-A. RFS was estimated at 26.1 months for the full cohort and 18.59 months for the elderly cohort.

Regarding toxicity, neutropenia was the most common side effect in both treatment groups and was observed in the vast majority (90%) of our patients receiving HMA-venetoclax, in accordance with previously reported real-world data [24]. Anemia and thrombocytopenia were also very common, at a rate of 85% and 67.5%, respectively, slightly less than some previous real-world reports [24], but higher than notified in VIALE-A [7]. Although retrospectively documenting real-life data is less accurate than randomized trials, partially contributing to these differences, it is obvious from our and previous data that hematologic toxicity is one of the main handling issues during HMA-venetoclax treatment in AML.

Comparing toxicity in patients treated with HMA monotherapy, even though patients treated with the HMA-venetoclax combination presented neutropenia more commonly (*p* = 0.020), infection rate and febrile neutropenia were similar in the two groups (*p* = 0.501 and *p* = 0.322, respectively). Febrile neutropenia was observed in 67.5% of patients in a HMA-venetoclax treatment setting, a higher percentage than previously notified [7,8,24]. GCSF was commonly used, especially with HMA-venetoclax, even in patients who were not in CR, despite conflicting data in the literature [15,17]. This observation highlights the prolonged character of neutropenia, complicating everyday clinical management. Dose delays occurred in the majority of our HMA-venetoclax-treated patients, which resulted in discontinuation of the drug in 20% of our patients, rates higher than previously reported [9,14]. The above results underlies the hematological toxicity rates in our cohort. 

Initial dose ramp-up was conducted in 87.5% of the patients, more than previous real-world data reports [9,12]. However, even with the use of cytoreduction and a ramp-up dosing schedule, TLS was presented in four patients (10%). Taking into consideration that TLS was lethal in one of these patients, we underline the need for TLS awareness. 

The infection rate in our cohort was 57.5%, higher than previously reported in a real-life setting of HMA-venetoclax use for AML [24,25]. Pneumonia was reported in 47.8% of the patients, a higher rate than in VIALE-A [7], but not higher than the historic control of HMA-monotherapy-treated patients in our centers. Moreover, invasive fungal infection (IFI) was noted in 3/40 patients (7.5%) in our combination cohort. Similar rates have been reported in patients without receiving any prophylaxis [8], while, on the other hand, a recent real-world study with 80% use of prophylaxis presented a 12.6% rate of IFI [26]. It is worth noting that only half of our patients received antifungal prophylaxis, and only 10% of our cohort received the gold standard of posaconazole for chemotherapy treatment of de novo AML [27,28]. Moreover, severe hepatotoxicity due to concomitant posaconazole use was observed in 1/40 patients, confirming previous concerns [18]. Recent European guidelines question the role of posaconazole in patients treated with HMA-venetoclax and propose further studies, including therapeutic drug monitoring, to determine the role of dosage adjustment of novel agents during concomitant administration of CYP3A4-inhibiting antifungals [29]. 

Moreover, even though the concerns of underexposure to venetoclax with lower doses remain, lower dosing or CYP3A4/P-gp inhibitor use was neither related to shorter OS nor RFS. Those results are in accordance with a post-hoc analysis of VIALE-A that demonstrated no impact on response or OS rates with the use of CYP3A inhibitors [30]. On the contrary, definite discontinuation of venetoclax predicted shorter OS and RFS (p_OS_ = 0.006, p_RFS_ = 0.01), indicating a possible benefit from reduced or delayed dosing rather than discontinuation of venetoclax treatment. Lastly, in a recent study at Mayo Clinic [14], the adverse ELN risk group was demonstrated to be a predictor of shortened survival. Even though high ELN risk stratification was not proven to have a prognostic value for OS in our cohort, a predictive value for shorter RFS (*p* = 0.021) was shown. This result indicates the possible contribution of adverse ELN risk to AML relapse in HMA-venetoclax-treated patients.

To conclude, even though it encompasses retrospective analysis constraints, our study reflects the trends and the everyday challenges of HMA-venetoclax use as a first-line treatment for AML patients. Confirming its approval study results, the addition of venetoclax to HMA treatment in AML patients unfit for intensive chemotherapy yields high response rates in a real-life setting and has shifted the treatment paradigm to chemotherapy-free options. Moreover, high rates of neutropenia and consequent infection render everyday management challenging for the clinician. Lastly, even though CYP3A4-inhibiting antifungals are recommended in AML patients undergoing induction chemotherapy, their role in HMA-venetoclax-treated patients is still controversial.

## Figures and Tables

**Table 1:** Baseline patient characteristics.

	HMA, *n* = 17 (100%)	HMA/VEN, *n* = 40 (100%)
Age, median (range)	76 (64–86)	73 (52–87)
Female sex	6/17 (35)	26/40 (65)
Karnofsky score median (range)	50 (20–100)	90 (60–100)
CIRS-G score median (range)	6.5 (4–12)	8 (4–12)
CYP3A4 Inhibitor Use:		
Strong	3 (17.6)	2 (5)
Moderate	3 (17.6)	7 (17.5)
Leukocytosis ≥ 25 × 10^9^/L	3 (17.6)	9 (22.5)
Anemia (Hb < 10 mg/dL)	16 (94.1)	35 (87.5)
Thrombocytopenia (PLTs < 100.000/μL)	12 (70.5)	25 (62.5)
Peripheral Blood Blast (%) median (range)	29 (1–68)	17 (0–90)
Bone Marrow Blast (%) median (range)	38 (20–77)	40 (20–90)
Karyotype:	N = 8	N = 35
Favorable	5 (62.5)	16 (45.7)
Intermediate	1 (12.5)	7 (20)
Adverse	2 (25)	12 (34.2)
Not Available	9	5
Mutations	N = 8	N = 36
FLT3 mutated	2 (25)	4 (11.1)
NPM1 mutated	2 (25)	9 (25)
Other mutations:	N = 3	N = 14
DNMT3A	2(66.6)	4 (28.5)
TET2	1 (33.3)	4 (28.5)
IDH1/2	0	3 (21.4)
TP53	1(33.3)	1 (7.1)
ASXL1	0	3 (21.4)
RUNX1	0	3 (21.4)
Not Available	14	26
ELN 2017 Risk Group:	N = 5	N = 34
High	1 (25)	13 (38.2)
Intermediate	3 (60)	16 (47)
Low	1 (25)	5 (14.7)
Not Available	12	6
TLS risk:	N = 15	N = 35
High	1 (5.8)	2 (5)
Intermediate	3 (17.6)	8 (20)
Low	13 (76.4)	25 (62.5)

**Table 2:** Hematologic and non-hematologic toxicity.

	HMA, *n* = 17 (100%)	HMA/VEN, *n* = 40 (100%)	*p*-Value
Hematologic:			
Anemia	10 (58.8)	34 (85)	0.092
Grade ¾	10 (58.8)	15 (37.5)	0.092
Neutropenia	11 (64.7)	36 (90)	0.020
Grade ¾	11 (64.7)	34 (85)	0.125
Thrombocytopenia	10 (58.8)	27 (67.5)	0.477
Grade ¾	9 (52.9)	18 (45)	0.331
Non-hematologic:			
Infection	9 (52.9)	23 (57.5)	0.501
Febrile Neutropenia	9 (52.9)	27 (67.5)	0.322
TLS	1 (5.8)	4 (10)	0.528

**Table 3:** Response evaluation.

	HMA, *n* = 17 (100%)	HMA/VEN, *n* = 40 (100%)
Cycles received [median (range)]	5 (1–61)	6 (1–35)
Venetoclax final dose		
400 mg		30 (75)
200 mg		5 (12.5)
100 mg		5 (12.5)
Cytoreduction	3 (17.6)	8 (20)
Time to first assessment in months [median (range)]	4.8 (1.8–13.2)	1.08 (0.3–10.1)
Response	Ν = 13	Ν = 38
Composite complete response (CCR)	4 (30.8)	33 (86.8)
Partial response (PR)	1 (7.6)	0 (0)
No response (NR)	7 (53.8)	5 (13.1)
Not available	4	0
Relapse	6 (35.3)	9 (22.5)
Number of cycles at relapse [median (range)]	4 (1–61)	6 (1–17)
Mortality	16 (94.1)	14 (35)
Due to progressive disease	5 (31.3)	7 (50)
Due to infection	10 (62.5)	6 (42.9)
Other reasons	1 (7.1)	1 (7.1)

**Table 4:** Univariate analysis of risk factors for OS and RFS.

Risk Factors	OS *p*-Value	RFS *p*-Value
Sex (male, female)	0.374	0.241
Karnofsky score (>80, ≤80)	0.128	0.193
CIRS-G (>6, ≤6)	0.805	0.833
Strong CYP3A4 inhibitor use	0.720	0.769
Moderate CYP3A4 inhibitor use	0.728	0.561
Posaconazole use	0.656	0.457
P-glycoprotein inhibitor use	0.295	0.837
Bone marrow blast percentage (>50%, ≤50%)	0.794	0.689
ELN risk group (high, low, or intermediate)	0.185	0.021
Anemia at diagnosis (Hb < 10 g/dL)	0.501	0.853
Leukocytosis at diagnosis (WBC ≥ 25 × 10^9^)	0.539	0.221
Thrombocytopenia at diagnosis (<100 × 10^9^/L)	0.159	0.471
High LDH at diagnosis (>225 IU/L)	0.256	0.343
Venetoclax dose (400 mg or less)	0.431	0.844
Delay or reduction of venetoclax	0.324	0.119
Definite discontinuation of venetoclax	0.006	0.010

## Data Availability

Data will be made available upon request to the corresponding author.
